# Hydroxychloroquine facilitates autophagosome formation but not degradation to suppress the proliferation of cervical cancer SiHa cells

**DOI:** 10.3892/ol.2014.1879

**Published:** 2014-02-13

**Authors:** QINGSONG LIU, XIONG YAN LUO, HONG JIANG, MING-HUI YANG, GUO-HUA YUAN, ZHONG TANG, HE WANG

**Affiliations:** 1 Key Laboratory of Obstetric and Gynecologic and Pediatric Diseases and Birth Defects of Ministry of Education, West China Institute of Women and Children’s Health, West China Second Hospital, Sichuan University, Chengdu, Sichuan 610041, P.R. China; 2 Institute of Rheumatology and Immunology, The Affiliated Hospital of North Sichuan Medical College, Nanchong, Sichuan 637000, P.R. China; 3 Department of Clinical Laboratory, The Affiliated Hospital of North Sichuan Medical College, Nanchong, Sichuan 637000, P.R. China

**Keywords:** hydroxychloroquine, cervical cancer, autophagy, apoptosis

## Abstract

Hydroxychloroquine (HCQ), the hydroxylated analog of chloroquine, is an antimalarial lysomotropic agent that inhibits autophagy due to lysosomal acidification, and subsequently blocks the fusion of autophagosomes with lysosomes which leads to the accumulation of autophagosomes that may accelerate tumor cell death. Given these hypothesis the aim of this study was to investigate the effects of HCQ in the inhibition of autophagy and the induction of apoptosis in cervical cancer SiHa cells. Cervical cancer SiHa cells were cultured with Hank’s balanced salt solution (HBSS) as positive control of autophagy or treated with HCQ as part of the experimental groups. LC3 and P62/SQSTM1 were detected by quantitative polymerase chain reaction (qPCR) and western blotting, respectively in order to evaluate initially autophagosome formation and their degradation. Specific green fluorescent protein (GFP)-LC3 was subsequently detected by fluorescence microscopy in order to confirm the formation of autophagosomes. MTT and flow cytometry were adopted respectively to assess the proliferation and apoptosis of the SiHa cells. miRNA-9* was also investigated. The results demonstrated that HCQ increased the expressions of LC3 mRNA and LC3II protein and GFP-LC3 signalling but reduced the expression of p62/STSQM1 in cervical cancer SiHa cells. These results indicated HCQ has the ability to inhibit autophagy as incapable of degrading the autophagosome. However, HCQ may promote SiHa cell apoptosis as the MTT, apoptotic assay and miRNA-9* results revealed. HCQ has the ability to inhibit autophagy by blocking the degradation of autophagosomes and subsequently facilitates the apoptosis of cervical cancer SiHa cells.

## Introduction

Cervical cancer is the second most common type of cancer and remains one of the most important causes of cancer-related mortality in females worldwide (1). The age of onset of cervical cancer has declined in recent years despite advances in radical surgery cures for the majority of early stage cervical cancer cases. Studies are now focusing on methods to improve the efficacy of treating locally advanced cervical cancer. Autophagy inhibition is at the forefront of cancer therapy. The initial interest in autophagy inhibition as a cancer therapy was generated by previous studies revealing that some cancers depend on autophagy for survival during external stresses, such as hypoxia, chemotherapy or radiotherapy (2).

Autophagy is commonly used to describe several distinct cellular processes during which long-lived proteins or cytoplasmic organelles are transported into the lysosomes for degradation (3). Hydroxychloroquine (HCQ), the hydroxylated analog of chloroquine, has antimalarial lysosomotropic agents that inhibit autophagy. Its involvement in the inhibition of autophagy is due to the affect of lysosomal acidification, followed by blocking the fusion of autophagosomes with lysosomes (4,5). Thereby, HCQ inhibits endogenous protein degradation, resulting in an increase in the number of autophagic compartments (6). Subsequently, HCQ treatment leads to the accumulation of autophagosomes that may accelerate tumor cell death. Since there is evidence that HCQ has a potential application in cancer treatment for its inhibition of autophagy in leukemic (7), breast cancer MCF-7 (8) and melanoma cells (9) and may subsequently induce their apoptosis, this process may also be important to the actions of HCQ in cervical cancer cells. Considering these observations, the present study sought to investigate the effects of HCQ in the autophagy inhibition of cervical cancer SiHa cells, thereby, exploring a novel therapeutic mechanism for this commonly used drug in cervical cancer.

## Materials and methods

### Cell culture and grouping

The human cervical cancer SiHa cell line was obtained from the Type Culture Collection of Wuhan University (Wuhan, China). The cells were grown in Dulbecco’s modified Eagle’s medium (Sigma-Aldrich, St. Louis, MO, USA) supplemented with heat-inactivated 10% fetal bovine serum (Gibco-BRL, Carlsbad, CA, USA), penicillin (100 U/ml; Sigma-Aldrich) and streptomycin (100 μg/ml; Sigma-Aldrich) at 37°C with 5% CO_2_. Following 24 h, cells were aspirated and washed with cold phosphate-buffered saline (PBS). Then, fresh medium, Hank’s balanced salt solution (HBSS, 100%; Thermo Fisher Scientific, Waltham, MA, USA) and HCQ (20μmol/l; Shanghai Ruiqi Biological Technology Co., Ltd., Shanghai, China) were added and cells were incubated for 1, 6, 12 and 24 h.

### Quantitative (q) polymerase chain reaction (PCR) analysis

Following incubation, total RNA from SiHa cells was extracted using the TRIzol total RNA extraction reagent (Tiangen Biotech (Beijing) Co., Ltd., Beijing, China) according to the manufacturer’s instructions. RNA concentration and purity was measured by ultraviolet spectrophotometry (Biodropsis BD-2000, Beijing Oriental Science and Technology Development Co., Ltd., Beijing, China). A total of 1 μg RNA was used to synthesize cDNA using SuperScript^®^ III reverse transcriptase (Invitrogen Life Technologies, Carlsbad, CA, USA) according to the manufacturer’s instructions. qPCR was performed on 2 μl of cDNA, 0.25 μl of each primer (10 pmol/μl) and 10 μl of SYBR Green master mix [Tiangen Biotech (Beijing) Co., Ltd., Beijing, China] to obtain a final reaction volume of 20 μl. The following primers were used for detection: 5′-AAT CCC GGT GAT CAT CGA GC-3′ and 5′-GCC GGA TGA TCT TGA CCA AC-3′ for human LC3 (Bioneer, Shanghai, China); and 5′-TCC CTG GAG AAG AGC TAC GA-3′ and 5′-AGC ACT GTG TTG GCG TAC AG-3′ for human β-actin [Sangon Biotech (Shanghai) Co., Ltd., Shanghai, China]. The primer ID used for the detection of human P62/SQSTM1 was no. 8,878 (Bioneer). The results were analyzed using ABI Prism software (Applied Biosystems, Carlsbad, CA, USA) and β-actin was used as an internal standard. The fold difference in cDNA abundance (F) was calculated using the following formula: $\text{F}=2^{-(\Delta \text{Ct}1-\Delta \text{Ct}2)}$, where ΔCt1 and ΔCt2 are the number of cycles required to reach the threshold of amplicon abundance for experimental and control conditions, respectively. All qPCR reactions, including no-template controls, were run using the ABI7900TH Fast Real-Time PCR system (Applied Biosystems) and performed in triplicate. Specificity of amplification of each transcript was confirmed by melting curve analysis using Sequence Detection System software (Applied Biosystems).

### Western blot analysis

Following culture, the attached SiHa cells were washed twice with PBS, harvested in PBS and then centrifuged to thoroughly remove the supernatant liquid. The amount of sediment suspended in the protein lysate (Beijing Biomed, Beijing, China) was according to the weight of the SiHa cells. In total, 20 μg of protein was loaded on a 15% sodium dodecyl sulfate polyacrylamide gel and transferred to polyvinylidene fluoride membranes. Following blocking, membranes were incubated for 1 h with anti-LC3 antibody or P62/SQSTM1, followed by a horseradish peroxidase-conjugated anti-rabbit IgG antibody (all Cell Signaling Technology, Inc., Danvers, MA, USA). Specific bands were detected by Enlight™ chemiluminescence reagents (Engreen Biosystem, Beijing, China). Anti-GAPDH (Cell Signaling Technology, Inc.) was used as an internal standard to ensure equal loading. Band intensity was semi-quantified using Fusion Fx5 software following imaging (Fusion Fx5, Vilber Lourmat, France).

### Fluorescence microscopy

Cells were plated on sterile coverslips and cultured under the conditions indicated. Once the cells reached 80% confluence, they were aspirated and washed with cold PBS. Then, fresh medium, HBSS and HCQ were added and cells were cultured for an additional 1 and 12 h. Green fluorescenct protein (GFP)-LC3 assays were performed using FlowCellect™ GFP-LC3 Reporter Autophagy Assay kit (Millipore, Billerica, MA, USA) with the following modifications. Next, 10 μl autophagy reagent A was added and cells were incubated in a humidified incubator at 37°C with 5% CO_2_ for 2 h. Media was aspirated and cells were washed with 5 ml 1X HBSS. Then, 100 ml 1X autophagy reagent B was added for 5 min, followed by one wash with 1X assay buffer to remove the 1X autophagy reagent B. The coverslips locked on the slides were rapidly removed and the slides were observed under an Olympus fluorescence microscope (BX51, Olympus Corporation, Tokyo, Japan).

### MTT assay for drug sensitivity

The sensitivity of the cells to starvation and HCQ was detected using MTT assay. Cells (5,000) were cultured in each well in a 96-well plate for 24 h. The culture medium was replaced with the medium containing serial dilutions of various chemotherapeutic drugs. Following 48 h of drug incubation, 20 μl 3-(4, 5- dimethylthiazol-2-yl)-2, 5-diphenyltetrazolium bromide (MTT; 5 mg/ml; Sigma-Aldrich) was added to each well and incubated for an additional 4 h. The supernatant was then removed and dimethyl sulfoxide (150 μl/well) was added to dissolve the blue formazan crystals converted from MTT by live cells. The absorbance (A) values of formazan were measured on a Tecan infinite M200Pro (Tecan, Männedorf, Switzerland) at 490 nm. The inhibition rate of tumor cells was calculated using the following formula: $\text{Inhibition rate}=[({\text{A}}_{\text{control}}-{\text{A}}_{\text{blank}})-({\text{A}}_{\text{treated}}-{\text{A}}_{\text{blank}})]/({\text{A}}_{\text{control}}-{\text{A}}_{\text{blank}})\times 100$. Each group was measured in triplicate wells on the same plate in three independent experiments.

### Flow cytometry analysis of cell apoptosis

Following incubation, the cells were simultaneously stained with fluorescein isothiocyanate (FITC)-labeled Annexin-V and propidium iodide (PI), according to the manufacturer’s instructions for the Annexin V-FITC apoptosis detection kit (Nanjing KeyGen Biotech Co., Ltd., Nanjing, China). A total of 1.0×10^6^ stable SiHa cells were washed twice with ice-cold PBS and incubated for 10 min in a binding buffer (including 5 μl PI and 5 μl Annexin V-FITC). Fluorescence-activated cell sorting analysis for Annexin-V and PI staining was performed by flow cytometry, to discriminate apoptotic cells (high FITC and low PI signals; and high FITC and high PI signals) and dead cells (low FITC and high PI signals) from viable cells (low FITC and low PI signals). All experiments were performed in triplicate.

### miRNA analysis

A stem-loop reverse transcription (RT)-PCR was adopted to validate the mature miRNA expression using SYBR Green PCR master mix (Applied Biosystems). The primers used for the detection of miRNA-9^*^ (primer ID, hsmq-0049) were purchased from GeneCopoeia (Rockville, MD, USA). Normalization was performed with U6 (primer ID, mRNA U6; GeneCopoeia). miRNA assays were performed using GeneCopoeia All-in-One miRNA qRT-PCR detection kit (cat. no. AOMD-Q020) as described previously. The formula used to calculate the relative expression of miRNA-9^*^ was as described previously for the qPCR.

### Statistical analysis

All statistical analyses were performed using SPSS 17.0 software (SPSS, Inc., Chicago, IL, USA). Studies were performed in triplicate and data are expressed as the mean ± standard deviation as appropriate. P<0.05 was considered to indicate a statistically significant difference, obtained by one-way analysis of variance (ANOVA) and least significant difference tests.

## Results

### HCQ induces the formation of autophagosomes of cervical cancer SiHa cells

**Figure 1:**
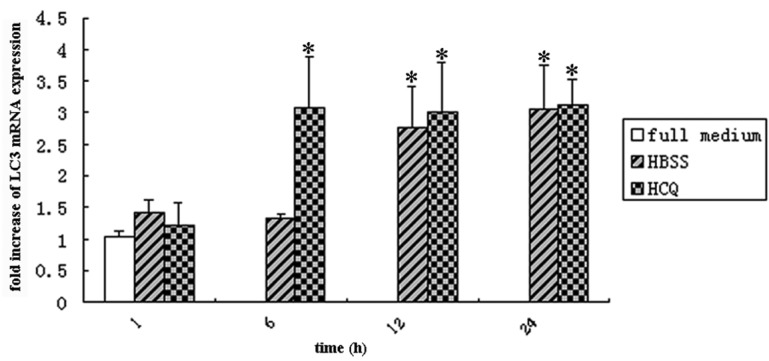
Expression of LC3 mRNA in cervical cancer SiHa cells treated with HBSS or HCQ. SiHa cells were treated with 20 μmol/l HCQ or starvation for 1, 6, 12 and 24 h. The relative expression of LC3 mRNA was detected by real-time polymerase chain reaction. Data are presented as the mean ± standard deviation (n=3). ^*^P<0.05 vs. full medium control. HBSS, Hank’s balanced salt solution; HCQ, hydroxychloroquine.

**Figure 2:**
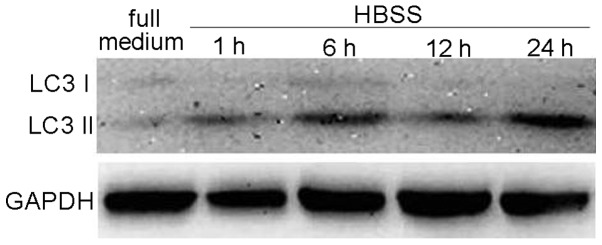
Expression of LC3 protein in cervical cancer SiHa cells treated with HBSS. Western blot analysis for the expression of LC3 protein in SiHa cells starved for 1, 6, 12 and 24 h. HBSS, Hank’s balanced salt solution.

**Figure 3:**
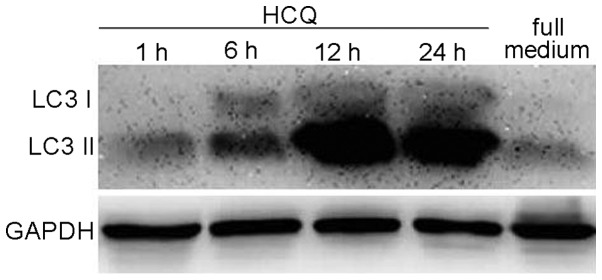
Expression of LC3 protein in cervical cancer SiHa cells treated with HCQ. Western blot analysis for the expression of LC3 protein in SiHa cells treated with 20 μmol/l HCQ for 1, 6, 12 and 24 h. HCQ, hydroxychloroquine.

Autophagy may be upregulated in response to amino acid starvation (10). Therefore, the nutrients of SiHa cells were withdrawn as the positive control of autophagy. LC3 acts as a marker to study the progression of autophagy in mammalian cell types. The qPCR results showed that the expression of LC3 mRNA was increased following starvation and HCQ stimulation (Fig. 1). During starvation, soluble LC3 (LC3-I) was cleaved by ATG4 and modified by the addition of a lipid, to form LC3-II (11). Western blot analysis showed the autophagosome marker, LC3-II, as the positive control (starvation; Fig. 2). LC3-II was also increased following HCQ treatment in cervical cancer SiHa cells (Fig. 3).

**Figure 4:**
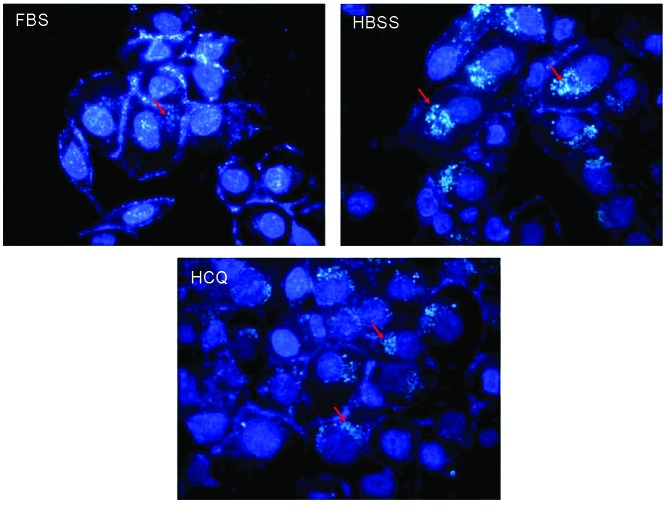
Specific GFP-LC3 combined fluorescence microscopy in cervical cancer SiHa cells treated with HBSS or HCQ. Cells were treated with 20 μmol/l HCQ and starvation for 12 h and stained with specific green fluorescent protein-LC3. Cell morphology was observed by fluorescence microscopy (red arrows indicate autophagosomes or LC3 accumulation). FBS, fetal bovine serum; HCQ, hydroxychloroquine; HBSS, Hank’s balanced salt solution.

To assess whether HCQ visually promotes autophagosome formation in cervical cancer SiHa cells, autophagosomes were stained with a specific GFP-LC3 Reporter Autophagy Assay kit and the presence of autophagosomes in SiHa cells was determined by fluorescence microscopy. The number of cells with autophagosomes and autophagosomes per cell was increased upon HCQ treatment and starvation (Fig. 4).

### HCQ inhibits autophagy by preventing autophagosome degradation in cervical cancer SiHa cells

**Figure 5:**
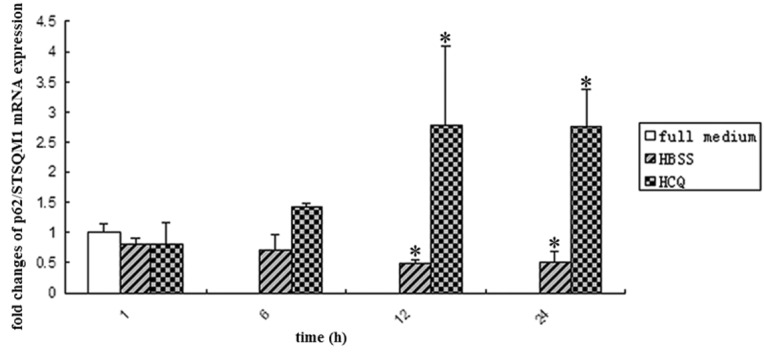
Relative expression of P62 mRNA in SiHa cells. SiHa cells were treated with 20 μmol/l HCQ and starvation for 1, 6, 12 and 24 h. The relative expression of LC3 mRNA was detected by real-time polymerase chain reaction. Data are presented as the mean ± standard deviation (n=3). ^*^P<0.05 vs. full medium control. HBSS, Hank’s balanced salt solution; HCQ, hydroxychloroquine.

**Figure 6:**
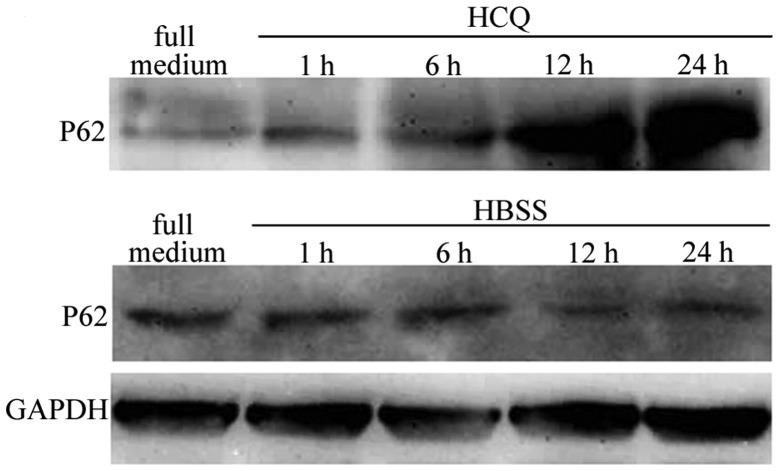
Relative expression of protein in SiHa cells. SiHa cells were treated with 20 μmol/l HCQ and starvation for 1, 6, 12 and 24 h. Western blot analysis was used to determine the LC3 protein expression in SiHa cells. HCQ, hydroxychloroquine; HBSS, Hank’s balanced salt solution.

During late autophagy, autophagosomes are delivered to lysosomes for degradation. HCQ is a potent autophagy inhibitor that affects lysosomal acidification and, thereby, inhibits endogenous protein degradation (6). P62/SQSTM1 is a ubiquitin- and LC3-binding protein that is known to regulate the degradation of targeted proteins via autophagy (12). P62/SQSTM1 is sequestered within autophagosomes and then degraded by lysosomes. The current study detected the levels of P62/SQSTM1 mRNA and protein with qPCR and western blot analysis, respectively. The results showed that the expression of P62/SQSTM1 mRNA and protein decreased in cervical cancer SiHa cells when starved for an increasing duration, but increased in HCQ-treated cells (Figs. 5 and 6).

### HCQ inhibits SiHa cell proliferation by inducing apoptosis

**Figure 7:**
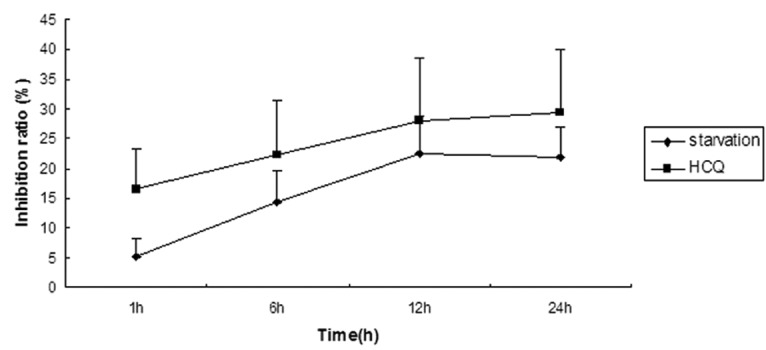
HCQ inhibits the proliferation of cervical cancer SiHa cells. SiHa cells were treated with 20 μmol/l HCQ or starvation for 1, 6, 12 and 24 h. Cell viability was determined by 3-(4, 5- dimethylthiazol-2-yl)-2, 5-diphenyltetrazolium bromide assay. Data are presented as the mean ± standard deviation (n=3). ^*^P<0.05 vs.. full medium control. HCQ, hydroxychloroquine.

**Figure 8:**
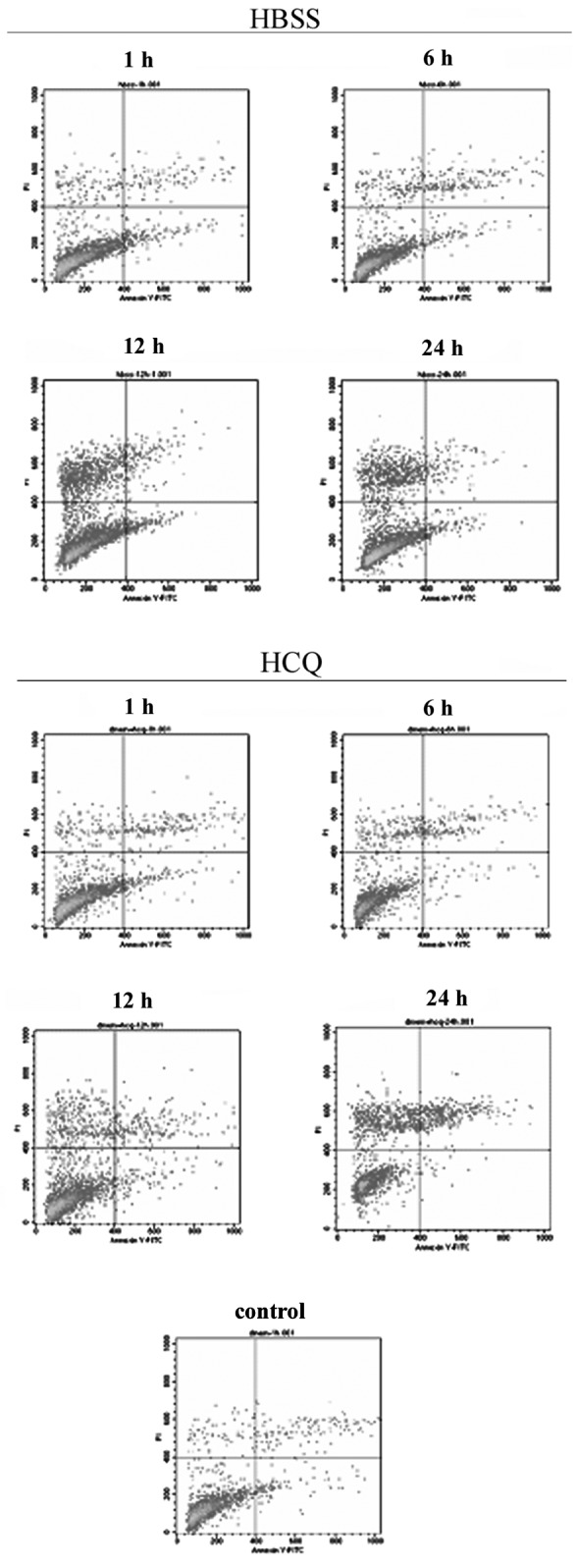
Flow cytometry detection of apoptotic and dead cells induced by HCQ or HBSS. SiHa cells were treated with 20 μmol/l HCQ and starvation for 1, 6, 12 and 24 h. HCQ, hydroxychloroquine; HBSS, Hank’s balanced salt solution.

**Figure 9:**
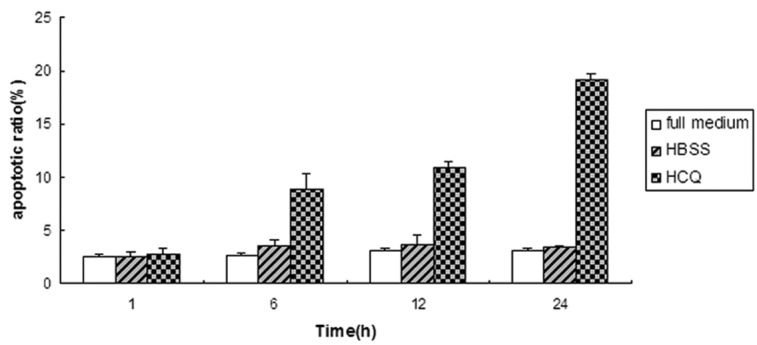
Quantitation of apoptotic cell ratio. Data are presented as the mean ± standard deviation (n=3). ^*^P<0.05 vs. full medium control. HCQ, hydroxychloroquine; HBSS, Hank’s balanced salt solution.

Autophagy may be a form of programed cell death or may be involved in cytoprotective activity in situations of nutrient starvation (13). Previously, Ramser *et al* found that HCQ significantly reduces the metabolic activity and suppresses the cell proliferation of human dermal fibroblasts (14). Therefore, the present study investigated whether HCQ suppresses cervical cancer SiHa cell proliferation. MTT results showed that the cell proliferation is inhibited when treated with HCQ and following starvation (Fig. 7). The flow cytometry results showed that the apoptotic ratio did not change significantly following starvation, but increased significantly following HCQ treatment (Figs. 8 and 9). The number of dead cells markedly increased following starvation and HCQ treatment for 12 and 24 h (Fig. 8).

**Figure 10:**
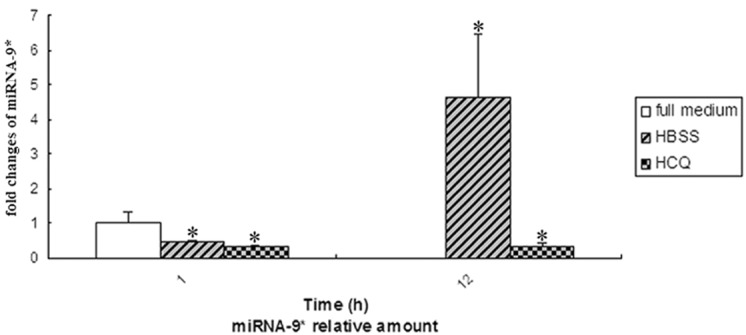
Relative expression of miRNA-9^*^ in cervical cancer SiHa cells treated with HBSS or HCQ. miRNA-9^*^ levels detected by stem-loop reverse transcription PCR with SYBR Green PCR master mix in SiHa cells treated with 20 μmol/l HCQ and starvation for 1 and 12 h. Data are presented as the mean ± standard deviation (n=3). ^*^P<0.05 vs. full medium control. HCQ, hydroxychloroquine; HBSS, Hank’s balanced salt solution; PCR, polymerase chain reaction.

miRNAs are involved in the modulation of a wide range of biological processes, including apoptosis and autophagy (15). miRNA-9* induces autophagic cell death in Waldenström macroglobulinemia (WM) cells (16). The levels of miRNA-9^*^ were detected by stem-loop RT-PCR. The results showed that the expression of miRNA-9^*^ was decreased following cell starvation for 1 h and treatment with HCQ for 1 and 12 h, but markedly increased following 12 h of starvation compared with the full medium control (Fig. 10).

## Discussion

HCQ is a potent autophagy inhibitor that affects lysosomal acidification and, thereby, inhibits endogenous protein degradation, resulting in an increase in the number of autophagic compartments (6). Preclinical studies have demonstrated that HCQ, the widely used antimalarial and antirheumatic drug, is a potent inhibitor of autophagy in cancer and increases tumor cell death alone or through enhancing tumor killing in combination with cytotoxic chemotherapy or targeted agents, mostly in patients with solid tumors (17), with the exception of cervical cancer. To the best of our knowledge, the current study provides the first evidence that HCQ inhibits the autophagy of cervical cancer SiHa cells and then prevents their proliferation by promoting apoptosis.

Autophagy involves the formation of autophagosomes that assemble around and encapsulate macromolecules, damaged organelles or cellular debris and then fuse with lysosomes to degrade their contents (18). Autophagosome detection may be based on LC3 (ATG8). This protein is first cleaved by ATG4 to generate LC3-I and is then lipidated to produce LC3-II (19). The lipidated (LC3-II) form may be detected as a faster-migrating band by immunoblotting (20). Simultaneously, the present study detected the mRNA and protein expression of LC3 by qPCR and western blot analysis, respectively. The results showed that the levels of LC3 mRNA and protein, similar to the positive control of autophagy (starvation), were increased significantly in SiHa cells treated with 20 μmol/l HCQ for 6, 12 and 24 h. As LC3-II is incorporated into the inner and outer surfaces of autophagosomes, the expression of a GFP-LC3 fusion protein may be used to identify GFP puncta or dots representing autophagosomes. Therefore, the current study also detected the GFP puncta with specific GFP-LC3 combined fluorescence microscopy and the results of the HCQ treatment group were similar to those of the positive control of autophagy (starvation). These results indicated that HCQ may be able to facilitate the autophagosome formation.

One critical point is that autophagy is a highly dynamic, multi-step process. However, the abovementioned approaches (LC3 lipidation on a western blot analysis and fluorescent GFP-LC3 dots) suffer from the limitation of a static measurement, rather than a measurement of the process (such as autophagosome degradation). An elevation of GFP-LC3 puncta or LC3-II levels may, for example, reflect the induction of autophagy, reduction in autophagosome turnover or the inability of turnover to keep pace with increased autophagosome formation but block the process at the level of degradation (6). Thus, while these assays provide useful information, additional results are required to confirm autophagy activation. P62/SQSTM1 exhibits multiple critical functions and is ultimately degraded by autophagy, therefore, serially monitoring levels of p62/SQSTM1 may also be considered a relative measure of flux (21). Notably, it was found that the protein of p62/SQSTM1 in SiHa cells was decreased following prolonged starvation, while increased following treatment with 20 μmol/l HCQ.

Autophagy is essential for survival when cells are faced with metabolic stress. However, the cell viability was inhibited by starvation and HCQ as detected by MTT in the present study. In contrast to the involvement of HCQ in survival-promoting activity, autophagy can be a form of programed cell death in situations of nutrient starvation. Prolonged stress and progressive autophagy may also eventually lead to cell death (22). Excessive cellular damage may lead to cell death by over stimulating autophagy and cellular self-consumption. The current study demonstrated that the dead cell ratio was markedly increased following starvation for 12 and 24 h. Autophagy functions as a survival mechanism and prevents apoptosis (23). Since it was observed that HCQ abrogates autophagy in cervical cancer SiHa cells, the present study determined whether HCQ treatment is likely to result in increased apoptosis of these cells as a consequence of impaired autophagy. The flow cytometry results showed that the apoptotic cells increased significantly following HCQ treatment for 6, 12 and 24 h versus the full medium control and starvation.

miRNA-9^*^, as a tumor suppressive miRNA, induces autophagic cell death in WM cells, by downregulating histone deacetylase (HDAC) 4 and HDAC5 and upregulating acetyl-histone-H3 and -H4 (16). In addition, the current study examined the levels of miRNA-9^*^ in cervical cancer SiHa cells following starvation for 1 and 12 h or treatment with HCQ for 1 and 12 h by stem-loop RT-PCR. The results showed that the levels of miRNA-9^*^ were increased significantly in SiHa cells following starvation for 12 h (in which autophagic cell death occurred) but decreased significantly in the other three groups (in which cell death or apoptosis did not occur).
